# Gut microbiota dysbiosis and joint diseases: advances in the gut-joint axis

**DOI:** 10.3389/fimmu.2026.1911852

**Published:** 2026-08-07

**Authors:** Haitao Li, Guojuan Li, Junzheng Liu

**Affiliations:** 1 Department of Joint Surgery, The Affiliated Nanhua Hospital, Hengyang Medical School, University of South China, Hengyang, Hunan, China; 2 Department of Endocrinology, The Affiliated Nanhua Hospital, Hengyang Medical School, University of South China, Hengyang, Hunan, China

**Keywords:** gut microbiota, gut-joint axis, immunity, joint diseases, metabolism

## Abstract

In recent years, the proposal of the “gut-joint axis” concept has provided a new perspective for understanding joint diseases. Research indicates that gut dysbiosis can drive the onset and progression of various joint diseases, including osteoarthritis, rheumatoid arthritis, spondyloarthritis, and gouty arthritis, through multiple pathways such as mediating systemic immune inflammation, disrupting metabolic homeostasis, and increasing intestinal permeability. This article systematically reviews the impact of specific alterations in the gut microbiota on different joint diseases and their underlying mechanisms, summarizes these shared and disease-specific mechanisms, and discusses novel diagnostic and therapeutic strategies targeting the microbiota, aiming to open new avenues for the prevention and treatment of joint diseases.

## Introduction

The human body is a complex ecosystem composed of human cells and symbiotic microorganisms, often referred to as a “super-organism” (1). In recent years, with the development of technologies such as metagenomics and metabolomics, research on the gut microbiota has made significant breakthroughs. People have gradually realized that gut microorganisms not only participate in the digestion and absorption of food, but also play a crucial role in physiological processes such as immune regulation, metabolic balance, and neuroendocrine regulation (2). The homeostasis of the gut microbiota is of great significance for maintaining human health. The normal gut microbiota inhibits the growth of pathogenic bacteria through various means such as spatial competition, nutritional competition, contact inhibition, and the secretion of antibacterial peptides and inhibitory metabolites (such as bile acids and short-chain fatty acids) (3); it produces short-chain fatty acids (SCFAs) such as acetic acid, propionic acid, and butyric acid by fermenting dietary fibers, providing energy for intestinal epithelial cells and maintaining the integrity of the intestinal barrier (4); it regulates the differentiation of immune cells such as regulatory T cells (Treg) and Th17 cells to maintain immune balance (5); it also participates in the metabolism of substances such as bile acids and tryptophan, affecting the overall energy metabolism and inflammatory state (6). However, when affected by genetic, dietary, antibiotic use, stress, and lifestyle factors (such as smoking and drinking), the gut microbiota may become imbalanced, with reduced microbiota diversity, while pathogenic bacteria and pro-inflammatory factors increase, leading to impaired intestinal barrier function, abnormal immune regulation, and metabolic disorders, and thereby participating in the occurrence and development of various diseases.

Studies have shown that dysbiosis of the gut microbiota is closely associated with various diseases, including digestive tract disorders [such as inflammatory bowel disease (7)], metabolic disorders [such as diabetes (8)], neurological disorders [such as autism (9)], cardiovascular diseases [such as atherosclerosis (10)], and autoimmune diseases [such as multiple sclerosis (11)]. In particular, in recent years, with the rapid development of microbiome technology, the close association between the gut microbiota and joint diseases has become a research hotspot (12). The concept of the “Gut-Joint Axis” has gradually emerged, providing a new perspective for understanding the pathogenesis of joint diseases and developing new treatment strategies.

Joint diseases are a common type of disorder with a high rate of disability. They mainly include osteoarthritis (OA), rheumatoid arthritis (RA), spondyloarthritis (SpA), and gouty arthritis (GA), etc (13). All of these significantly affect the quality of life and working ability of patients. For a long time, OA has been regarded as a local mechanical wear disease, mainly related to mechanical damage to the articular cartilage and imbalance in the repair process (14); RA is considered to be an inflammatory disease caused by abnormal activation of the immune system, leading to synovial inflammation and joint destruction (15); SpA is viewed as an inflammatory disease related to genetics and immune abnormalities, which can affect both the axial and peripheral joints and may present with extra-articular manifestations (16); GA is caused by dysregulation of purine metabolism, resulting in hyperuricemia, and subsequently the deposition of monosodium urate crystals, leading to episodic and severe inflammatory joint diseases (17). Based on these understandings, current treatment approaches mainly focus on symptom relief (such as non-steroidal anti-inflammatory drugs), immunosuppression (such as DMARDs), and joint function replacement (such as joint replacement surgery). However, there is currently a lack of treatment methods to reverse disease progression, and the current treatment effects are often unsatisfactory. Early studies have found that an increase in the relative abundance of *Streptococcus species* can lead to knee joint pain and an increase in the severity of OA (18). The microbial composition of patients with early rheumatoid arthritis showed significant changes compared to the control group. Specifically, the numbers of *Bifidobacterium* and *Bacteroides* decreased (19). After targeted drug treatment, the dysregulated intestinal microbiota will partially return to normal (20). Moreover, a study in 1958 showed that ulcerative colitis has a potential association with ankylosing spondylitis (AS) (21). Therefore, researchers began to turn their attention to the “intestinal-joint axis”, attempting to open up a new path for disease intervention by regulating the intestinal microecology.

**Figure 1:**
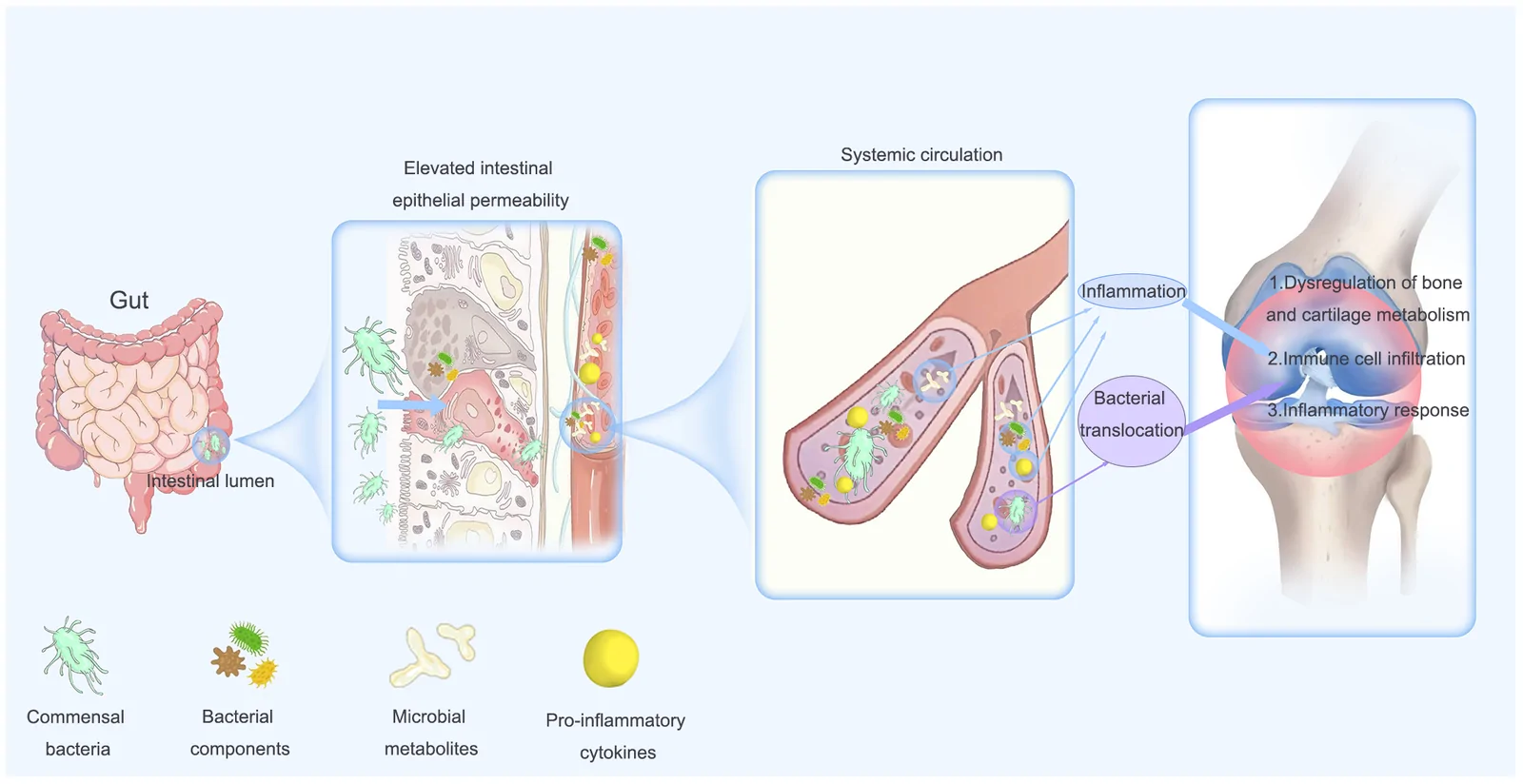
Schematic diagram illustrating the mechanisms by which gut microbiota dysbiosis contributes to joint pathology (This figure was created entirely by the authors and the copyright belongs to the authors).

In recent years, with the deepening of research, the concept of the “gut-joint axis” has gradually emerged and has been supported by experimental evidence. Existing evidence shows that the intestinal microbiota and its metabolites can affect joint health through multiple pathways: first, through immune regulation, dysbiosis of the microbiota can lead to systemic immune imbalance, promoting the production of inflammatory factors and attacking joint tissues (12, 22); second, through metabolites, the SCFAs, bile acids, and tryptophan metabolites produced by the microbiota can enter the circulation system and regulate the inflammatory state and metabolic balance of the joints (23); third, through the intestinal barrier, dysbiosis of the microbiota can lead to increased intestinal permeability, allowing bacterial components (such as LPS) to translocate into the blood circulation and activate the systemic immune response (24); fourth, through bacterial translocation, bacteria or their components may migrate to the local joints, locally regulating immune cell infiltration and bone metabolism balance (25) (**Figure 1**). These studies not only push the intestinal microecology to the core pathogenic link of joint diseases, but also provide new directions for the development of new treatment strategies. Therefore, this article provides a narrative review of the specific changes of the intestinal microbiota in different joint diseases such as osteoarthritis, rheumatoid arthritis, spondyloarthritis, and gouty arthritis, summarizes its mechanism of action, and looks forward to the research direction and clinical translation potential of the treatment strategies based on the microbiome.

### Gut microbiota and osteoarthritis

Osteoarthritis is the most common chronic degenerative joint disease, characterized by degradation of joint cartilage, remodeling of subchondral bone, formation of bone spurs, and varying degrees of synovial inflammation. Traditional studies have mainly focused on local mechanical abnormalities and the cellular metabolic disorders and imbalance of inflammatory factor regulation of tissues such as cartilage, synovium, and subchondral bone. In recent years, with the proposal of the “gut-joint axis” concept, the role of the gut microbiota in the occurrence and development of OA has gradually attracted attention.

**Figure 2:**
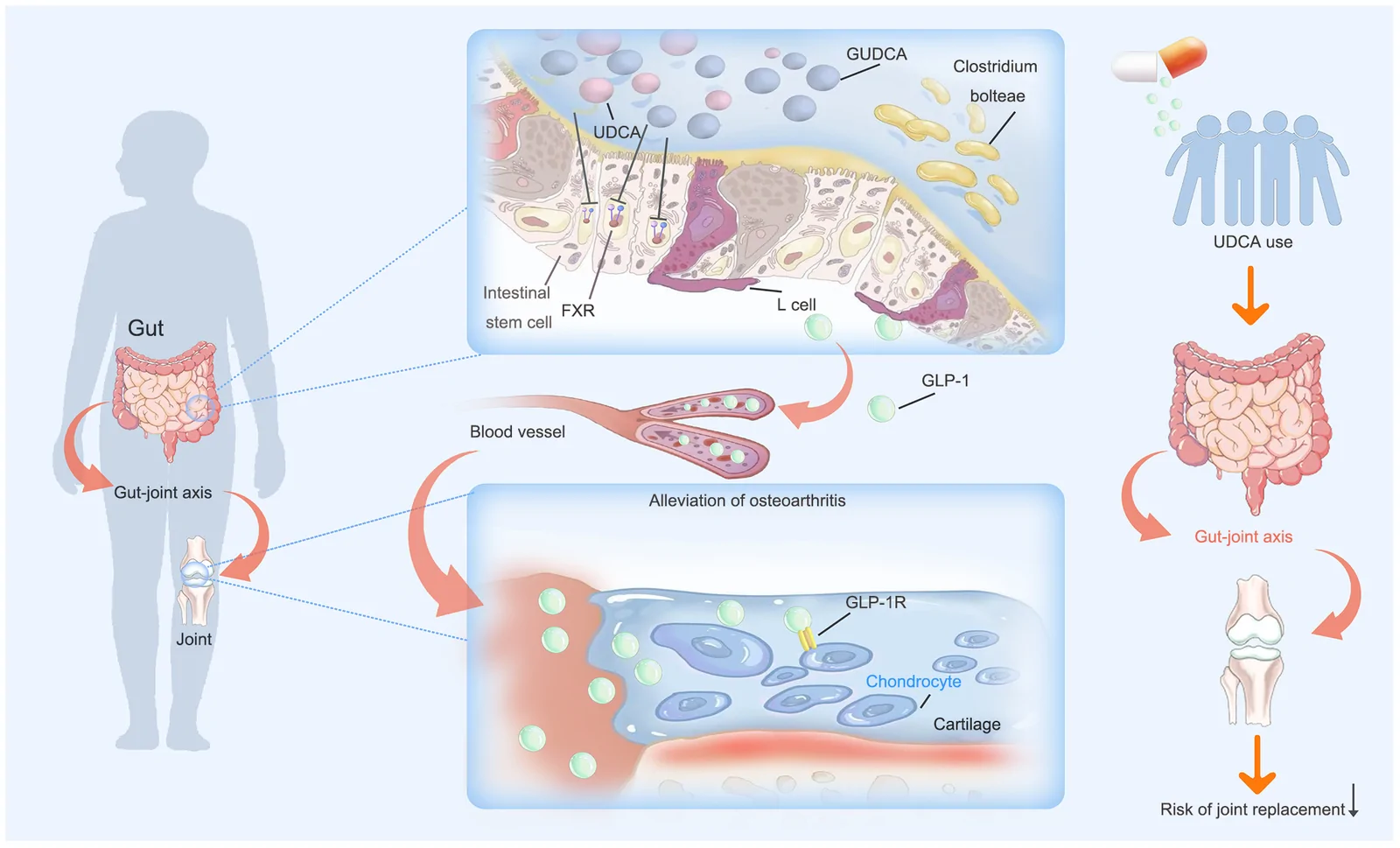
Schematic diagram illustrating the regulation of the osteoarthritis progression by the “gut microbiota - bile acid - FXR - GLP-1 axis” (Adapted and redrawn from reference 26 with modifications. The original source is cited in the reference list).

**Figure 3:**
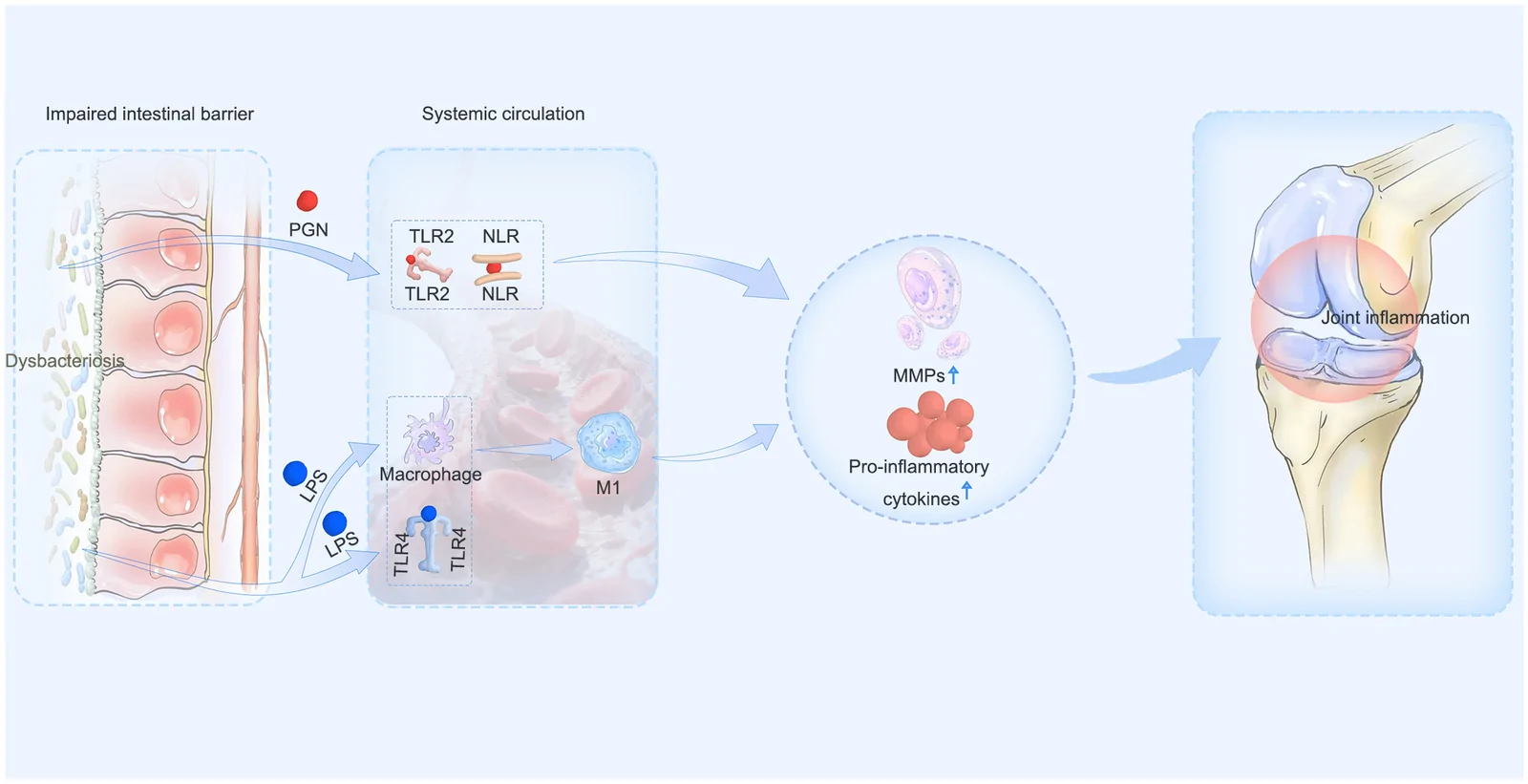
Schematic diagram illustrating the mechanism by which intestinal flora imbalance activates systemic and joint inflammation through disrupting the intestinal barrier function (This figure was created entirely by the authors and the copyright belongs to the authors).

In 2025, the pioneering research conducted by Lei Guanghua’s team revealed the crucial role of the “gut microbiota - bile acid - FXR - GLP-1 axis” in the pathogenesis of OA (26). Through metagenomic sequencing of the feces of 981 individuals, the study found that the diversity of the gut microbiota in OA patients was significantly reduced, the abundance of commensal bacteria such as *Clostridium bolteae* (C. bolteae) decreased, and it was positively correlated with the level of glycoursodeoxycholic acid (GUDCA). The dysbiosis of the gut microbiota in OA patients led to abnormal bile acid metabolism, with a decrease in the level of GUDCA, which then activated the intestinal farnesoid X receptor (FXR) and inhibited the secretion of GLP-1; while GLP-1 acts on the GLP-1 receptor (GLP-1R) in the joints through the bloodstream, which can inhibit cartilage degradation. Supplementing GUDCA or colonizing *C. bolteae* could inhibit the FXR signal, increase the secretion of GLP-1, and significantly alleviate the progression of OA in mice (**Figure 2**). Apart from the bile acid pathway, intestinal flora imbalance leads to the weakening of the intestinal barrier and an increase in its permeability, allowing the microorganisms in the intestine and their metabolite LPS to enter the systemic circulation (27). In this process, bacterial peptidoglycans (PGN) and LPS act as ligands that bind to pattern recognition receptors, thereby initiating an inflammatory response. Specifically, PGN engages Toll-like receptor 2 and nucleotide-binding oligomerization domain-like receptors (NLRs), leading to the induction of matrix metalloproteinase (MMP) expression, inflammasome activation, and upregulation of pro-inflammatory factors (24, 28). In addition, LPS can directly drive macrophages in the innate immune system to differentiate into the M1 phenotype. By forming complexes with Toll-like receptor 4, LPS stimulates these macrophages to produce pro-inflammatory cytokines, including IL-1β, TNF, and MMPs. This process leads to pronounced secondary inflammation in joint tissues as well as systemic effects (29, 30) (**Figure 3**).

At present, there have been many studies on regulating intestinal flora imbalance by increasing the abundance of beneficial bacteria, alleviating inflammatory responses, and slowing down the progression of OA. A double-blind randomized controlled trial published in 2024 showed that Multistrain probiotics enhanced the functional ability of OA patients (31). Jhun et al. found that oral *Lactobacillus rhamnosus* could inhibit inflammation and thereby slow down the development of OA (32).

However, there are still many shortcomings in the current research. Firstly, although the “bile acid-FXR-GLP-1 pathway” has been initially clarified, whether the gut microbiota affects OA through other metabolites or signaling pathways (such as short-chain fatty acids (33), tryptophan metabolism) still requires further exploration. Moreover, most current studies are cross-sectional or animal experiments, and there is a lack of longitudinal cohort studies to clarify whether gut microbiota imbalance is the initiating factor of OA or a consequence of joint lesions. Secondly, the clinical evidence for microbiota intervention is limited. The existing clinical trials have small sample sizes, short intervention periods, lack of unified strains, doses, and treatment protocols, and the efficacy and safety still need to be verified by large-scale multicenter studies. The research on individualized differences also has significant deficiencies. The composition of the gut microbiota is affected by various factors such as genetics, diet, region, and drugs. Currently, there are no individualized microbiota intervention strategies for different OA phenotypes or different populations, which makes it difficult to promote current research. Finally, the role of the “brain-gut axis” in OA is gradually attracting attention, but how the gut microbiota affects the central regulation of pain and the local inflammatory microenvironment of the joint through the vagus nerve or neuroendocrine pathways remains unclear, and related research is almost nonexistent.

### Gut microbiota and rheumatoid arthritis

Rheumatoid arthritis is a chronic autoimmune disease characterized by synovial inflammation, joint swelling, pain, and progressive joint destruction. Studies have shown that the genetic probability of RA varies among different populations, with the HLA-DRB1 gene being the main risk factor (34). However, even those carrying high-risk genes, most people will not develop the disease, indicating that genes are not the sole determining factor. Currently, the widely accepted view is that a multi-factor pathogenic cascade reaction involving genetics, environment, and the microbiome promotes the occurrence and development of RA. The “gut-joint axis” as a new concept has gradually become a research hotspot in the field of RA.

**Figure 4:**
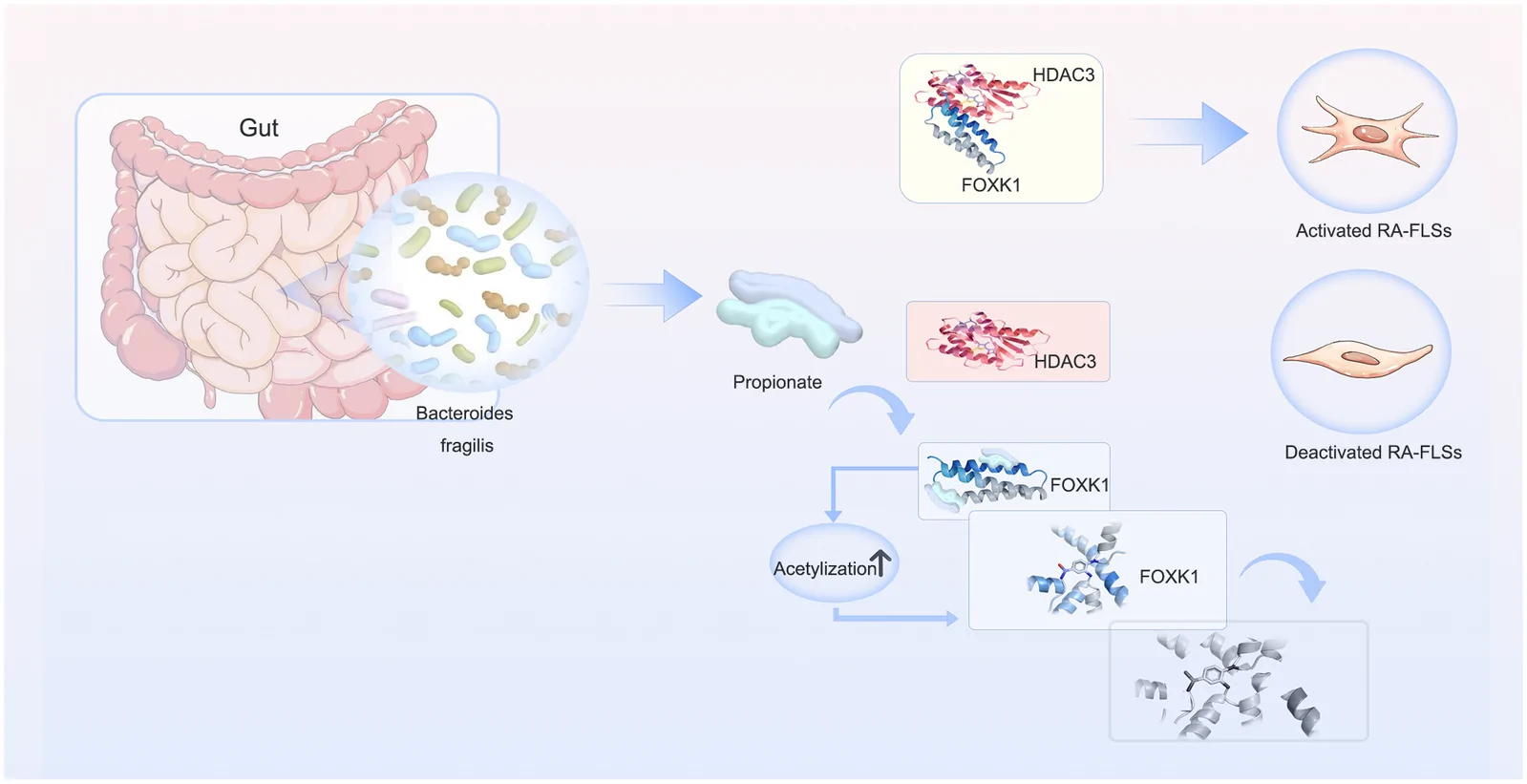
Schematic diagram illustrating the mechanism by which propionate derived from *Bacteroides fragilis* inhibits RA through the “HDAC3-FOXK1-interferon axis” (Adapted and redrawn from reference 35 with modifications. The original source is cited in the reference list).

In 2024, Chen et al. demonstrated in mouse models and human samples that propionate produced by *Bacteroides fragilis* can inhibit RA through the “HDAC3-FOXK1-interferon axis”. Mechanistically, propionate blocks the binding of HDAC3 and FOXK1, enhances the acetylation of FOXK1 and reduces its stability, thereby inhibiting the interferon signaling pathway, suppressing the proliferation, invasion and secretion of inflammatory factors of fibroblast-like synoviocytes (FLSs), and delaying joint destruction. The authors also proposed a microbiota-directed therapy centered on *B. fragilis* or propionate, which can serve as a supplement or alternative to existing immunosuppressive regimens, providing a new strategy for the targeted treatment of RA targeting FLSs (35) (**Figure 4**). In terms of the composition of the microbiota, Alpizar-Rodriguez et al. reported that the abundance of *Prevotella* was higher and that of *Bacteroide*s was lower in preclinical RA patients (36). As early as 2014, it was discovered that propionate could reduce the activity of Th2 effectors by activating the GPR41 receptor expressed in dendritic cells, increase IL-10 levels, promote Treg differentiation, and exert immunomodulatory effects (37). Another key short-chain fatty acid - butyrate - through inducing Treg polarization, down-regulating pro-inflammatory cytokines and inhibiting the production of autoantibodies, plays a preventive role in the development of RA (38). In the intestines of RA patients, the bacteria *Faecalibacterium* and *Roseburia* that produce butyrate were significantly reduced. Supplementing these strains can restore anti-inflammatory signals, inhibit the activity of traditional Th cells, and thereby control systemic inflammation (39). Multiple species of *Lactobacillus* (such as *Lactobacillus reuteri*, *Lactobacillus casei*, *Lactobacillus rhamnosus*, *Lactobacillus acidophilus*, *Lactobacillus brevis*, *Lactobacillus salivarius* and *Lactobacillus fermentum*) have also been proven to alleviate inflammation and relieve arthritis (40). Among them, *Lactobacillus plantarum* and *Lactobacillus salivarius* can increase the activity of Treg cells and down-regulate the activity of Th17 cells; *Lactobacillus rhamnosus* and *Lactobacillus fermentum* mainly inhibit Th17 immune responses; *Lactobacillus reuteri* and *Lactobacillus casei* mainly focus on inhibiting Th1 immune responses, and work together through multiple pathways to reduce joint inflammation (41). In the pathogenic process, when sterile Sakai Koyasu Granulocyte (SKG) mice were implanted with fecal samples rich in *P. copri* from RA patients, intestinal Th17 cells significantly proliferated and severe arthritis occurred, suggesting that this bacterium may play an important role in driving the disease (42). Zhao et al. demonstrated that *E. coli* and *S. bovis* promote the onset and progression of RA by enhancing the degradation of ascorbic acid (43). Parisa Ahmadi further summarized that *Porphyromonas gingivalis* promotes the pathogenesis and progression of RA by degrading proteins via its gingipains and mediating citrullination through PPAD, while simultaneously inducing NETosis to release host PAD enzymes, thereby enhancing the production of citrullinated antigens that are recognized by Anti-Cyclic Citrullinated Peptide Antibody (ACCPAs) (44). At the mechanism level, microbial peptides have a high affinity for the HLA-DRB1 “shared epitope” molecules, which is the most important genetic risk factor for anti-citrullinated peptide antibody (ACPAs)-positive RA (45). Additionally, molecular mimicry is also considered to be the key for RA initiation. Pianta et al. discovered that N-acetyl-glucosamine-6-sulfatase (GNS) and Filamin A (FLNA) expression were elevated in the joint fluid and synovium of RA patients, both of which are self-antigens and can induce T cell auto-reactions in more than 50% of patients (46). The GNS protein has the same epitope sequence as the *Proteus mirabilis* and *Prevotella* proteins, while FLNA contains similar epitopes in the proteins of another intestinal symbiotic bacteria, *Bifidobacterium* and *Prevotella.* Therefore, *Prevotella* may trigger autoimmunity through molecular mimicry of GNS and FLNA, ultimately leading to the occurrence and progression of RA (46).

Published studies have confirmed that the probiotics *Faecalibacterium prausnitzii* and *Lactobacillus casei CCFM1074* can significantly inhibit the inflammatory response in RA model, alleviate joint tissue damage and reduce the arthritis score (41, 47). In addition to intervention through supplementing probiotics or microbial metabolites, strategies for reshaping the intestinal microbiota through specific diets and thereby improving the symptoms of rheumatoid arthritis are receiving increasing attention. Research shows that Bai et al. found that a high-fiber diet (HFD) rich in resistant starch (RS) can eliminate the increase in the abundance of lactobacilli and enterococci related to rheumatoid arthritis (48). The above results consistently indicate that specific dietary components can shape the structure of the intestinal microbiota, increase the proportion of butyrate-producing bacteria and anti-inflammatory bacteria, inhibit pro-inflammatory signals, providing experimental evidence for the dietary-microbiota combined intervention for RA.

Currently, research on gut microbiota and RA shares similar limitations with that on OA. However, it is noteworthy that, whereas the causal relationship between gut microbiota alterations and OA onset remains unclear, higher concentrations of zonulin (a precursor of haptoglobin known to induce leaky gut syndrome and increase intestinal permeability) have been observed in the serum of RA patients and collagen-induced arthritis mice (49). According to a study by Kinashi and Hase, upregulation of zonulin and increased intestinal permeability occur prior to the onset of arthritis, and treatment with a zonulin antagonist alleviated disease symptoms (50). This may suggest that leaky gut syndrome acts as an initial event in the rheumatoid arthritis disease cascade.

### Gut microbiota and spondyloarthritis

Spondyloarthritis is a group of immune-mediated diseases characterized by inflammation of the axial skeleton, peripheral joints, and attachment sites. It encompasses ankylosing spondylitis (AS), psoriatic arthritis (PsA), reactive arthritis (ReA), inflammatory bowel disease (IBD)-related arthritis, and a subgroup of juvenile idiopathic arthritis (51). Human studies suggest that the overall alteration of the gut microbiota, including decreased diversity, impaired barrier function, and abnormal specific microbial markers, may play a crucial role in the onset, progression, and management of SpA (52). Although the exact mechanism of SpA remains unclear, it is currently believed that genetic susceptibility (especially the presence of HLA-B27), intestinal barrier dysfunction, and environmental factors (such as exposure to specific microbiota) interact to trigger abnormal immune activation, thereby driving the occurrence and persistence of the disease (53).

**Figure 5:**
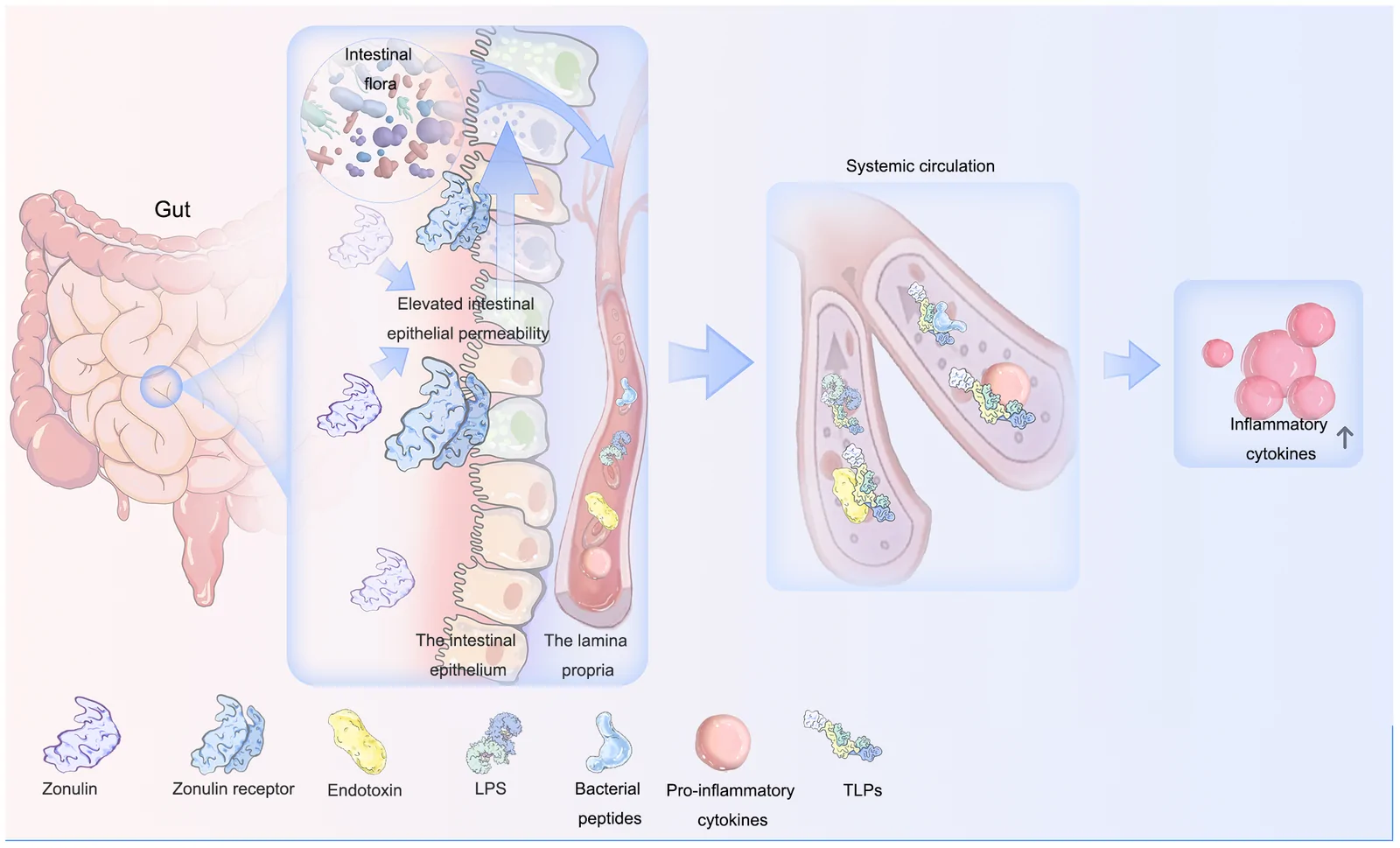
Schematic diagram illustrating the mechanism by which elevated zonulin levels lead to increased intestinal permeability, thereby inducing and maintaining chronic inflammation of spinal arthritis (This figure was created entirely by the authors and the copyright belongs to the authors).

The association between gut microbiota and SpA varies depending on the study. In axial SpA (axSpA), the abundances of *Ruminococcus gnavus (*53), *Dialister genus (*54), *E. coli (*55), and *Faecalibacterium prausnitzii (*56) are not only related to the disease, but in some cases, also related to disease activity. Compared to healthy individuals and psoriasis patients, the levels of *Ruminococcus* and *Akkermansia* in PsA patients are reduced (57). These bacteria do not exist in isolation; they affect the whole body through the “intestinal barrier-immune” axis. Gut barrier dysfunction and TLR-mediated inflammation, as detailed in the OA section, also contribute to SpA, but the process is further amplified in HLA-B27-positive individuals, driving the chronic inflammation typical of this disease (49, 58) (**Figure 5**). Clinical data support this pathway: more than half of axSpA patients exhibit microscopically evident gut inflammation despite the absence of overt IBD symptoms (59). Concurrently, their serum levels of zonulin, LPS, lipopolysaccharide-binding protein (LPS-BP), and intestinal fatty acid-binding protein (iFABP) are elevated. Immunohistochemical analyses further confirm downregulated expression of tight junction proteins, increased bacterial translocation, and aggravated inflammation in the lamina propria (60). The increase in α4β7+ T cells and MAIT cells within the synovium (61) suggests the homing of gut-derived immune cells to the joints. A similar immunological skewing is observed in PsA, characterized by significantly elevated levels of TH9, TH17, TH22 cells and IL-9 (62). Animal models have provided causal evidence: germ-free HLA-B27 transgenic rats did not develop arthritis. Following colonization with *segmented filamentous bacteria* (SFB) or *Prevotella*, Th17 cells were rapidly activated and spondylitis subsequently emerged, confirming that the “microbiota-Th17” axis constitutes a core circuit in SpA (63). Transgenic rats expressing HLA-B27 and human β2-microglobulin spontaneously developed ileitis and SpA-like arthritis; antibiotic treatment reversed this process: tight junctions were restored, bacterial translocation decreased, and inflammation in both the gut and joints was alleviated (60). It is noteworthy that the role of *Prevotella* is context-dependent, it can be pro-inflammatory, yet may also confer metabolic benefits in the context of a plant-based diet (64). Therefore, identifying specific bacterial strains directly associated with disease activity is more critical than mere taxonomic classification. Tito et al. were the first to identify a positive correlation between *Dialister* and disease activity (54); Berland et al. further confirmed the enrichment of *Dialister* and *Coprococcus* in HLA-B27-positive individuals, which was associated with higher disease activity and elevated levels of pro-inflammatory cytokines (65). Berland and colleagues also found that the gut microbiota is not only associated with disease activity but may also be strongly influenced by HLA-B27 status (65). Recent studies suggest that HLA-B27 itself can shape the microbiota: differences in gut microbial composition exist between HLA-B27-positive SpA patients and HLA-B27-positive healthy controls, indicating that genetic background influences SpA phenotypes via the microbiota (56).

In contrast to rheumatoid arthritis, the impact of high-fiber, plant-based, or other restrictive diets on gut microbiota diversity and inflammation in patients with SpA remains unexplored. This gap represents an attractive avenue for future research. A clear understanding in this area could provide a theoretical foundation and practical implementation strategies for the non-pharmacological management of SpA. Beyond diet, exercise also has a direct influence on the gut microbiota and, when appropriate, can help reduce inflammation and improve overall physical function (66). Promoting regular exercise combined with a balanced and tailored diet may help control SpA symptoms and enhance patients’ motivation to maintain physical activity, which is crucial for long-term disease management. Future studies are needed to further validate the potential of these lifestyle interventions as non-pharmacological therapies. If combined with conventional standard medications, they hold promise for reducing disease activity and promoting long-term health.

Current research on gut microbiota alterations in SpA still presents several significant shortcomings. First, exploration into metabolites remains relatively limited. For instance, studies have observed altered tryptophan metabolism in axSpA patients, increased 3-hydroxypropionic acid, decreased SCFAs, and altered bile acids in PsA patients. Most findings remain at the descriptive, correlative level, lacking mechanistic insights into how these metabolites specifically regulate immune responses through microbe-host interactions. In particular, there is insufficient experimental evidence for causality, making it difficult to determine whether metabolic changes are drivers or consequences of the disease. Secondly, microbiome studies themselves face notable limitations. The reported associations between gut microbes and SpA vary considerably across different studies, likely due to small sample sizes, population heterogeneity, or methodological inconsistencies, leading to challenges in reproducibility and generalizability. Although the abundance of *Ruminococcus gnavus*, *Dialister*, *E. coli*, and *Faecalibacterium prausnitzii* has been correlated with disease status and activity in axSpA, and reductions in *Ruminococcus* and *Akkermansia* have been noted in PsA, these findings are predominantly based on relative abundance analyses. They fail to elucidate the functional changes of specific bacterial strains or their precise roles within the “gut barrier–immune” axis. For example, while a positive correlation between *Dialister* and disease activity has been reported, its pro-inflammatory mechanisms and strain-specific functions remain unclear. Third, although the reshaping effect of genetic factors like HLA-B27 on the microbiota has been emphasized, the specific mechanisms underlying the genetic-microbial interactions that differentiate the microbiota of HLA-B27-positive and negative individuals are not well understood. Furthermore, the context-dependent roles of bacteria such as Prevotella, influenced by factors like diet, have not been sufficiently explored, resulting in an incomplete interpretation of microbial functions. Finally, current research predominantly focuses on bacterial taxonomy rather than functional genes or metabolic pathways. There is a marked insufficiency in studies targeting microbial strains and molecular biomarkers directly linked to disease activity. This gap significantly hinders the translation of these findings into clinical diagnostics and therapeutic strategies.

### Gut microbiota and gouty arthritis

Gouty arthritis is an acute inflammatory joint disease triggered by purine metabolism disorders leading to hyperuricemia, followed by the deposition of monosodium urate (MSU) crystals in joints and surrounding tissues (67). Traditionally, GA has been viewed as an immune and metabolic disorder. However, with the recent proposal of the “gut-joint axis” concept, the gut microbiota has emerged as a new research focus, believed to participate in the onset and recurrence of GA by modulating uric acid metabolism, inflammasome activation, and immune responses.

Studies indicate that gut microbiota dysbiosis, in conjunction with genetic and environmental factors, contributes to the onset and progression of GA (68). Chen et al. (69) analyzed fecal samples from 26 GA patients and 28 healthy individuals in China, revealing that the GA group exhibited enrichment of bacteria including *Escherichia coli*, *Bifidobacterium infantis*, *Blautia wexlerae*, and *Klebsiella pneumoniae*. In contrast, the healthy control group was predominantly characterized by the presence of *Faecalibacterium prausnitzii*, *Agathobacter rectalis*, and *Bacteroides*. Co-abundance network analysis uncovered complex interactions among multi-kingdom microbial features, suggesting their synergistic influence on GA and demonstrating promising potential for disease diagnosis. Gut barrier dysfunction and LPS/TLR4 activation, as described in the OA section, also contribute to GA (70); this pathway is further supported by animal models showing Proteobacteria expansion and LPS/TLR4/MyD88 upregulation in gouty geese (71). Furthermore, gut microbiota disruption affects uric acid excretion. The expression of major urate transporters, SLC2A9 and ABCG2, is decreased in GA patients, which is associated with impaired function of SCFAs produced by gut microbiota (72, 73). Aberrant SCFAs metabolism leads to reduced production of urate transporters and metabolic enzymes (such as hydrolases and uricase) by intestinal epithelial cells. This subsequently diminishes uric acid excretion, elevates serum uric acid levels, and promotes the deposition of MSU crystals, triggering subsequent immune-inflammatory responses (74–76). Of note, the uricase gene exists in humans but has been inactivated during evolution, resulting in a lack of functional uricase in humans (77). Additionally, SCFAs (such as acetate) can alleviate MSU crystal-induced inflammation by inducing neutrophil apoptosis (78). Some anti-gout medications may exert therapeutic effects by modulating the gut microbiota and promoting SCFA production (79). Gut microbes also directly participate in purine and uric acid metabolism: *Escherichia coli* and bacteria from the phylum *Proteobacteria* secrete xanthine dehydrogenase, promoting the conversion of purines to uric acid (80); *Lactobacillus* can inhibit intestinal purine absorption, preventing an increase in serum UA levels; *Pseudomonas* synthesizes uricase, contributing to uric acid degradation (81). Characteristic alterations in the gut microbiota of gout patients include an increase in *Prevotella*, *Clostridium*, and *Bacteroides*, alongside a decrease in *Enterobacteriaceae*, *butyrate-producing bacteria*, *Coprococcus*, and *Bifidobacterium* (82). The reduction in *Enterobacteriaceae* is associated with decreased functions in amino acid metabolism and environmental sensing, collectively contributing to elevated serum uric acid and C-reactive protein levels (83). Using stable isotope tracing technology, Liu et al. identified 46 uric acid-degrading bacterial strains from the human gut microbiota, belonging to phyla such as *Actinobacteria* and *Firmicutes*. These strains can metabolize uric acid to xanthine or SCFAs (84). Hyperuricemia model mice also exhibit decreased abundance of *Bifidobacterium* and *Lactobacillus*, accompanied by increased levels of uric acid, xanthine oxidase (XOD) activity, and LPS (85).

In summary, the gut microbiota and its metabolites play a central role in the pathogenesis and progression of GA by modulating inflammatory pathways and affecting uric acid synthesis and excretion. Furthermore, alterations in metabolites resulting from microbial dysbiosis (such as abnormal metabolism of polyphenols, vitamins, and tryptophan) may also contribute to disease pathogenesis (86).

Prebiotics, probiotics, fecal microbiota transplantation (FMT), and specific dietary interventions have emerged as important strategies for treating GA through the modulation of the gut microbiota. Prebiotics exert therapeutic effects by inducing the proliferation of beneficial bacteria, promoting the production of SCFAs, enhancing intestinal barrier function, regulating immune responses, and improving microbial structure. Probiotics, primarily represented by *Lactobacillus* and *Bifidobacterium*, have demonstrated dual potential in experimental studies, exhibiting both anti-inflammatory effects and the ability to reduce serum uric acid levels (87). Among the various strategies for regulating the gut microecology, research on Traditional Chinese Medicine (TCM) for the prevention and treatment of GA has garnered increasing attention. Multiple studies indicate that the underlying mechanisms of various TCM formulations in treating GA are closely associated with their precise regulation of the gut microbiota. For instance, Lin et al. found that Simiao Decoction effectively alleviated GA symptoms by modulating pro-inflammatory cytokine levels and repairing the gut ecosystem (88); Bian reported that Guizhi Shaoyao Zhimu Decoction ameliorated gouty arthritis in rats by altering the gut microbiota and improving metabolic profiles (89); furthermore, Wang et al. demonstrated that Modified Baihu Decoction significantly restored the abundance of beneficial bacteria such as *Lactobacillaceae* and *Bifidobacteriaceae*, thereby re-establishing microbial equilibrium (90). Collectively, these studies reveal that TCM exerts holistic regulatory effects via the “microbiota-metabolism-immunity” axis, providing novel perspectives and a scientific basis for GA treatment.

Although current research has significantly elucidated the central role of gut microbiota in the pathogenesis of GA, several limitations persist, warranting further exploration in future studies. Firstly, the majority of investigations remain predominantly cross-sectional and observational, making it difficult to definitively establish whether the observed microbial alterations are a cause or a consequence of GA. Secondly, the research on the mechanism is still at an early stage. Although key metabolites such as SCFAs and LPS, along with associated signaling pathways (such as TLR4/NF-κB), have been identified, their specific targets and detailed molecular cascades are not fully elucidated. Finally, regarding therapeutic translation, while prebiotics, probiotics, and TCM show considerable potential, their specific active components, optimal treatment combinations, and long-term efficacy and safety profiles require standardized evaluation. Translating microbial signatures into reliable clinical diagnostic tools and developing targeted micro-ecological therapies represent crucial goals for future research.

## Discussion

Through a narrative review of OA, RA, SpA, and GA, this study reveals that although these four articular disorders differ in clinical manifestations and pathological cores, they all exhibit profound and intricate associations with gut microbiota. Such connections are first reflected in a series of common mechanisms: Gut microbiota dysbiosis may serve as an initiating event for dysfunction of the “gut-joint axis”; subsequent intestinal barrier disruption and “leaky gut” facilitate the translocation of bacteria and their products, which acts as a key driver of systemic and local joint inflammation. In particular, the translocation of microbe-associated molecular patterns (MAMPs), exemplified by LPS, extensively exacerbates the inflammatory cascade in various types of arthritis through activation of innate immune receptors such as TLR4. Concurrently, dysregulation of SCFAs, which possess immunomodulatory and metabolic regulatory functions, along with the depletion of beneficial bacterial populations such as *butyrate-producing Faecalibacterium*, constitutes a common pathophysiological background. This suggests that restoring microbiota homeostasis and metabolic balance may represent a potential cross-disease management strategy. Nevertheless, each disorder also exhibits unique “microbiota-host” interaction patterns, which determine disease-specific initiation and progression trajectories. For example, the distinctive core mechanism of OA lies in the “gut microbiota (*C. bolteae*)-bile acid (GUDCA)-FXR-GLP-1 axis,” which directly influences cartilage protection. RA, in contrast, is characterized notably by molecular mimicry triggered by bacteria such as *Prevotella*, driving autoimmunity, as well as the regulation of synovial cell function via specific epigenetic pathways mediated by propionate derived from *Bacteroides fragilis.* The uniqueness of SpA resides in the robust interplay between the HLA-B27 gene and gut microbiota (such as *Dialister*), which constitutes the distinct genetic-microbiological basis of its pathogenesis. Meanwhile, the core specificity of GA lies in the direct involvement of gut microbiota in regulating the entire process of uric acid metabolism (**Table 1**). It is worth noting that the same gut bacterial species may play different roles in different diseases. *Prevotella* species have been reported to promote Th17-mediated inflammation and drive arthritis in RA (as shown in SKG mouse models), yet in SpA, their role appears context-dependent-Prevotella may be pro-inflammatory in certain settings, but may also confer metabolic benefits in the context of a plant-based diet. This duality highlights the complexity of interpreting microbial associations without considering dietary and environmental factors. *Escherichia coli* is enriched in the gut microbiota of GA patients and has been associated with increased xanthine dehydrogenase activity and uric acid production. In contrast, in SpA, *E. coli* has been linked to disease activity and gut inflammation, while in RA, *E. coli* has been implicated in promoting disease through ascorbic acid degradation. This suggests that the same bacterial species may exert disease-specific effects depending on the underlying immunological and metabolic context.

**Table 1:** Shared and disease-specific factors and mechanisms of gut microbiota in joint diseases.

Category	Factors/Mechanisms	Role and evidence in the four joint diseases
Shared Factors /Mechanisms	1.Gut microbiota dysbiosis	Alterations in microbial composition are observed in patients with OA, RA, SpA, and GA, representing a common foundation for “gut-joint axis” dysfunction (26, 35, 57, 69).
	2.Impaired intestinal barrier & “Leaky Gut”	All four diseases showed increased intestinal permeability (with elevated markers such as serum zonulin and LPS), leading to the translocation of bacteria and their products, which is a crucial step in triggering systemic and local joint inflammation (27, 49, 58, 70).
	3.Translocation of MAMPs	LPS is a core pathogenic factor that is mentioned in all four diseases. It activates receptors such as TLR4, driving the polarization of macrophages and the production of pro-inflammatory factors (such as IL-1β and TNF-α), thereby exacerbating joint inflammation (29, 49, 70, 94).
	4.Disorder SCFAs metabolism	SCFAs (especially butyrate and propionate) have demonstrated significant immunomodulatory and metabolic regulatory functions in RA, OA, SpA, and GA (33, 38, 74, 95).
	5.Depletion of beneficial bacteria	The reduction of the bacterium *Faecalibacterium* is a common feature (33, 39, 96, 97).
Disease-Specific Factors/Mechanisms	OA	Specific bacterium: Abundance of *Clostridium bolteae* correlates with GUDCA levels and OA severity (26). Core metabolic axis: The “Gut Microbiota-Bile Acid (GUDCA)-FXR-GLP-1 Axis” is a key OA specific pathway. Dysbiosis reduces GUDCA, activates intestinal FXR, suppresses GLP-1 secretion, and subsequently weakens the chondroprotective effect of GLP-1R (26).
	RA	Autoimmunity trigger: *Prevotella* can induce autoimmunity in ACPA-positive RA via molecular mimicry (e.g., mimicking autoantigens like GNS, FLNA) (46). Immune core imbalance: Th17/Treg imbalance and autoantibodies (RF, ACPA) are immunological cores distinguishing RA (38, 41). Bacterium-metabolite axis: Propionate derived from *Bacteroides fragilis* specifically suppresses the pathogenicity of fibroblast-like synoviocytes via the HDAC3–FOXK1–interferon axis, revealing a novel therapeutic target (35).
	SpA	Genetic-microbiota interaction: The HLA-B27 genotype strongly shapes gut microbiota, correlating with enrichment of specific taxa (such as *Dialister*), which are associated with disease activity, forming the unique genetic-microbiological basis of SpA (56, 65).
	GA	Direct regulation of urate metabolism: Gut microbiota directly participates in purine/urate synthesis, catabolism, and excretion, representing the most GA-specific mechanism. Key bacteria include: *Escherichia coli* (promotes urate production), *Pseudomonas* (secretes uricase to degrade urate), and *Lactobacillus* (inhibits purine absorption) (80, 81).

In-depth exploration of the “gut-joint” axis holds significant clinical implications. First, it provides novel biomarkers for the early diagnosis and prognostic assessment of joint diseases. For example, by analyzing a patient’s gut microbiota composition or specific serum metabolites, we may potentially predict their risk of developing arthritis or precisely evaluate disease activity. Second, it opens up new avenues for the treatment of joint diseases. Traditional therapeutic approaches primarily focus on suppressing local inflammatory responses within the joints. The “gut-joint” axis theory, however, suggests that modulating the gut microbiota to control systemic inflammation at its source may represent a more fundamental therapeutic strategy. For instance, employing probiotics, prebiotics, fecal microbiota transplantation, or specific dietary interventions to restore gut microbiota homeostasis holds promise as adjunctive or alternative therapies for arthritis. Furthermore, supplementing specific metabolites produced by the gut microbiota, such as propionate and butyrate, could also emerge as a novel treatment modality. These interventions based on the “gut-joint” axis are anticipated to lead to more personalized and precise treatment regimens for patients with arthritis.

However, translating this cutting-edge theory into established clinical practice still faces numerous challenges. The core bottleneck lies in confirming causality. Currently, most evidence supporting causality derives from animal models. In human studies, due to ethical and methodological constraints (such as the difficulty in directly observing the trafficking of cells between the gut and joints) the relevant evidence is predominantly observational, making it difficult to establish definitive causal pathways. This hinders the translation of mechanistic discoveries into clinical applications. To overcome these limitations, future research urgently needs to incorporate more well-designed prospective human cohorts and interventional clinical trials. Regarding therapeutic strategies, beyond existing tools like probiotics, more precise gut microbiota remodeling shows significant potential. For example, employing phages to selectively eliminate pro-inflammatory bacterial strains (91), using nano-oligosaccharide particles to precisely modulate specific microbial communities (92), or directly supplementing key metabolites to enhance anti-inflammatory effects (93). These innovative therapies aim to fundamentally restore gut micro-ecological balance, thereby achieving sustained and effective suppression of joint inflammation.

## Conclusion

The in-depth study of the “gut-joint” axis has opened up a new perspective for the diagnosis and treatment of joint diseases. Although the causal chain from dysbiosis of the microbiota to joint inflammation still needs to be further confirmed in the human body, strategies such as using prebiotics, targeting microbial remodeling, and metabolite intervention to restore the intestinal microecological balance undoubtedly lay a promising theoretical foundation for developing source-based and precise prevention and treatment plans for arthritis.
